# Therapeutic Implications of PPAR*γ* in Cardiovascular Diseases

**DOI:** 10.1155/2010/876049

**Published:** 2010-08-12

**Authors:** Hiroshi Hasegawa, Hiroyuki Takano, Issei Komuro

**Affiliations:** ^1^Department of Cardiovascular Science and Medicine, Chiba University Graduate School of Medicine, 1-8-1 Inohana, Chuo-ku, Chiba 260-8670, Japan; ^2^Department of Cardiovascular Medicine, Osaka University Graduate School of Medicine, 2-2 Yamadaoka, Suita, Osaka 565-0871, Japan

## Abstract

Peroxisome proliferator-activated receptor-*γ* (PPAR*γ*) is the members of the nuclear receptor superfamily as a master transcriptional factor that promotes differentiation of preadipocytes by activating adipose-specific gene expression. Although PPAR*γ* is expressed predominantly in adipose tissue and associated with adipocyte differentiation and glucose homeostasis, PPAR*γ* is also present in a variety of cell types including vascular cells and cardiomyocytes. Activation of PPAR*γ* suppresses production of inflammatory cytokines, and there is accumulating data that PPAR*γ* ligands exert antihypertrophy of cardiomyocytes and anti-inflammatory, antioxidative, and antiproliferative effects on vascular wall cells and cardiomyocytes. In addition, activation of PPAR*γ* is implicated in the regulation of endothelial function, proliferation and migration of vascular smooth muscle cells, and activation of macrophages. Many studies suggest that PPAR*γ* ligands not only ameliorate insulin sensitivity, but also have pleiotropic effects on the pathophysiology of atherosclerosis, cardiac hypertrophy, ischemic heart, and myocarditis.

## 1. Introduction

Cardiovascular disease (CVD) is the leading cause of morbidity and mortality in the western world. Major risk factors for CVD is hypertension, dyslipidemia, and hyperglycemia including insulin resistance [1]. Many antihypertensive drugs are widely used to normalize blood pressure, and its evidence of CVD preventive effect has been clarified [2]. Moreover, several classes of drugs for dyslipidemia, including bile acid sequestrants, fibrates, and statins, have been used historically to reduce cholesterol levels and reduced the morbidity and mortality of CVD [3]. Meanwhile, insulin has been used to improve hyperglycemia as a pivotal controller of basic glucose metabolism. Since the insulin self-injection of every day is intolerable [4], other agents that are used to help control elevated glucose concentrations in diabetes have been developed. These agents can decrease glucose production by the liver (metformin), stimulate insulin secretion (sulfonylureas and meglitinides), retard glucose absorption from the gastrointestinal tract (-glycosidase inhibitors), slow intestinal motility (amylin), reduce insulin resistance (thiazolidinediones (TZDs)), or replace gastrointestinal peptides known to be active in glucose metabolism (GLP-1 analogs or dipeptidyl peptase IV inhibitors). TZDs are a class of glucose-lowering oral medications which improve glycemic control and improve insulin sensitivity in muscle and liver [5]. TZDs exert their hypoglycemic properties by reducing insulin resistance through stimulation of a nuclear receptor, peroxisome proliferator-activated receptor *γ* (PPAR*γ*). PPAR*γ* is associated with adipocyte differentiation and glucose homeostasis. PPAR*γ* is expressed in a variety of cell types, including adipocytes, macrophages, vascular smooth muscle cells (VSMCs), endothelial cells (ECs), and cardiomyocytes [6–11]. Since it was reported that activation of PPAR*γ* suppresses production of inflammatory cytokines in activated macrophages, many researchers paid attention to PPAR*γ* as a new therapeutic target for CVD.

RXR, which interacts with the PPAR*γ*, is activated by 9-cis retinoic acid. When combined as a PPAR*γ*:RXR heterodimer, the PPAR*γ* ligands and 9-cis retinoic acid act synergistically on PPAR*γ* responses. The corepressor complex constitutes corepressor proteins, such as nuclear receptor corepressor (NCoR) and silencing mediator of retinoid and thyroid hormone receptors, histone deacetylases (HDACs) and transducin *β*-like protein 1 (TBL1). HDACs are essential in maintaining repressed chromatin structure and TBL1 exchanges a corepressor complex for a coactivator complex in the presence of ligand [12]. Many nuclear receptors are proposed to sequester inflammatory transcription factors, such as nuclear factor-*κ*B (NF-*κ*B) and AP-1, by inhibiting their DNA-binding activities, resulting in inhibition of inflammatory target genes. In the presence of ligand, PPAR*γ* also interacts with inflammatory transcription factors and inhibits their DNA-binding activities. PPAR*γ* blocks clearance of the corepressor complex in a ligand-dependent manner, and PPAR*γ* stabilizes the corepressor complex bound to the promoter of inflammatory genes [13]. It was demonstrated that PPAR*γ* associates with the protein inhibitor of activated STAT1 (PIAS1), which is a small ubiquitin-like modifier (SUMO)-E3 ligase, in a ligand-dependent manner. PIAS1-induced SUMOylation of the ligand-binding domain of PPAR*γ* enables the receptor to maintain NCoR on the promoter of inflammatory genes [14]. These are the suggested mechanisms of PPAR*γ* transrepression.

Activity of PPAR*γ* is depressed by phosphorylation of a serine residue (Ser112) in the N-terminal domain, mediated by a member of the mitogen-activated protein (MAP) kinase family, extracellular signal-regulated protein kinase (ERK). In addition, another member of MAP kinase family, c-Jun N-terminal kinase (JNK), also phosphorylates PPAR*γ* at Ser82 and reduces the transcriptional activity of PPAR*γ*. The association of PPAR*γ* polymorphism with metabolic syndrome has also been examined [15, 16]. In the presence of ligand, PPAR*γ* bind to coactivator complexes, resulting in the activation of target genes. In the absence of ligand, PPAR*γ* binds to the promoters of several target genes and associates with a corepressor complex, leading to active repression of target genes. This process is referred to as active repression.

Several ligands, which bind to PPAR*γ*, resulting in conformational change and activation of PPAR*γ*, have been discovered [17]. 15d-PGJ2, which is the PGD2 metabolite, was the first endogenous ligand for PPAR*γ* to be discovered. Although 15d-PGJ2 is the most potent natural ligand of PPAR*γ*, the extent to which its effects are mediated through PPAR*γ* in vivo remains to be determined. Two components of oxidized low density lipoprotein (ox-LDL), the 9-hydroxy and 13-hydroxy octadecadienoic acids (HODE), are also potent endogenous activators of PPAR*γ* [18, 19]. Activation of 12/15-lipoxygenase induced by interleukin (IL)-4 also produced endogenous ligands for PPAR*γ* [20], however, whether these natural ligands act as physiological PPAR*γ* ligands in vivo remains unknown. TZDs, such as troglitazone, pioglitazone, ciglitazone, and rosiglitazone are pharmacological ligands of PPAR*γ*. They bind to PPAR*γ* with various affinities and exerts insulin-sensitizing and hypoglycemic effects by activating PPAR*γ*. However, the molecular mechanisms by which TZDs affect insulin resistance and glucose homeostasis are not fully understood. They seem to mediate their effects primarily through adipose tissue, because TZDs alter the expression level of genes that are involved in lipid uptake, lipid metabolism and insulin action in adipocytes. TZDs enhance adipocyte insulin signaling and reduce the release of free fatty acids. TZDs also decrease the inflammation of adipose tissue that is induced by obesity and contributes to increased insulin resistance. TZDs improve insulin sensitivity in skeletal muscle and liver, which is the main insulin-sensitive organs, through these multiple adipocentric actions. TZDs has been demonstrated to have an anti-inflammatory effect, leading to initiation of treatment trials for patients with inflammatory diseases including CVD [21].

## 2. Role of PPAR*γ* in Atherosclerosis

Atherosclerosis is a chronic, complex and progressive pathological process in large- and medium-sized arteries. Atherosclerotic vascular disease is the most common cause of vascular complications, including stroke, myocardial infarction (MI), and aortic aneurysms/dissections. There are multiple potential mechanisms contributing to susceptibility to atherosclerosis. Injury of the endothelium, proliferation of VSMCs, migration of monocytes/macrophages, and the regulatory network of growth factors and cytokines are important in the development of atherosclerosis. Hypertension, dyslipidemia, increased free radicals from smoking and diabetes causes chronic inflammation of the vascular wall and abnormal immune response. Their formation is triggered by endothelial cell activation and dysfunction causing the release of vasoactive molecules and cytokines, which stimulate an inflammatory response and recruitment/migration of leukocytes into the arterial wall [22]. Increased expression of adhesion molecules such as vascular cell adhesion molecule-1 (VCAM-1), intracellular adhesion molecule-1 (ICAM-1), E-selectin and P-selectin within the atherosclerotic lesion stimulates monocyte recruitment and transmigration into the arterial intima [23], and accumulation of lipids and extracellular matrix may further amplify the local inflammatory response [24]. Monocytes rapidly mature into tissue macrophages which take up oxidized lipoproteins via scavenger receptors within the subendothelial space. Intracellular accumulation of cholesterol results in the characteristic formation of foam cells and stimulates macrophages to secrete cytokines, growth factors, and other mediators that promote smooth muscle cell proliferation and potentiate the inflammatory response, leading to arterial remodeling. A vicious circle of inflammation and cell infiltration causes plaque progression, which interfere with the normal blood flow. Eventually, the plaque ruptures due to degradation by macrophage-induced matrix metalloproteinases (MMPs) and hydrolytic enzymes, resulting in thrombus formation and tissue infarction [22].

**Figure 1:**
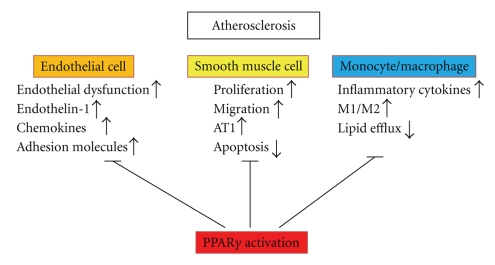
Action of PPAR*γ* on atherosclerotic lesion. PPAR*γ* act on endothelial cell, smooth muscle cell, and macrophage.

PPAR*γ* has anti-inflammatory effect and PPAR*γ* ligands have been shown to reduce production of inflammatory cytokines, such as IL-1*β*, IL-6, inducible nitric oxide synthase and tumor necrosis factor-*α* (TNF-*α*), by inhibiting the activity of transcription factors such as activator protein-1 (AP-1), signal transducers and activators of transcription (STAT), and NF-*κ*B in monocytes/macrophages [6, 7]. These findings suggest that PPAR*γ* activation may have beneficial effects in modulating inflammatory responses in atherosclerosis [25, 26]. Interestingly, expression of PPAR*γ* has been demonstrated in atherosclerotic plaques [25]. Macrophages affect the vulnerability of plaque to rupture, and they are implicated in the secretion of matrix metalloproteinases (MMPs), enzymes that are important in the degradation of extracellular matrix. In macrophages and VSMCs, PPAR*γ* ligands have been shown to reduce the expression of MMP-9, resulting in the inhibition of migration of VSMCs, and plaque destabilization [7, 8]. Although activation of T lymphocytes represents a critical step in atherosclerosis, PPAR*γ* ligands also reduce the activation of T lymphocytes [27]. T lymphocytes also express PPAR-*γ*, cells important in atherosclerosis, and can limit their chemokine elaboration [28]. Classically activated macrophages (M1) express a high level of proinflammatory cytokines and reactive oxygen species, whereas alternatively activated macrophages (M2) play an anti-inflammatory role in atherosclerosis. Recently, it was reported that PPAR*γ* is a key regulator of M1/M2 polarization [29]. PPAR*γ* agonists prime monocytes into M2 and PPAR*γ* expression is enhanced by M2 differentiation [30]. VSMC proliferation and migration are also critical events in atherosclerosis and vascular-intervention-induced restenosis. TZDs inhibit both these changes in the VSMCs and neointimal thickening after vascular injury [31–34]. Furthermore, TZDs induce apoptosis of VSMCs via p53 and Gadd45 [35, 36]. Angiotensin II (AngII) plays an important role in vascular remodeling via the AngII type 1 receptor (AT1R) and accelerates atherosclerosis. Although AngII induces transcriptional suppression of PPAR*γ*, activation of PPAR*γ* inhibits AT1R gene expression at a transcriptional level in VSMCs [37–39]. Expression of adhesion molecule by ECs, leading to adhesion of leukocytes, is a critical early step in atherosclerosis. PPAR*γ* ligands inhibit the expression of vascular cell adhesion molecule-1 (VCAM-1) and intercellular adhesion molecule-1 and decreased production of chemokines, such as IL-8 and monocyte chemotactic protein-1 (MCP-1) via suppressions of AP-1 and NF-*κ*B activities in ECs [40–42]. PPAR*γ* ligands also inhibit MCP-1-induced monocytes migration [43]. Endothelin-1 (ET-1) is involved in the regulation of vascular tone and endothelial functions, and induces proliferation of VSMCs. In bovine aortic ECs, PPAR*γ* ligands suppressed transcription of the ET-1 promoter by interfering with AP-1 [44]. PPAR*γ* activation by major oxidized lipid components of ox-LDL, 9-HODE and 13-HODE has an important role in the development of lipid-accumulating macrophages through transcriptional induction of CD36, a scavenger receptor. [45]. These findings suggest that atherogenic ox-LDL particles could induce their own uptake through activation of PPAR*γ* and expression of CD36, leading to atherosclerosis. However, several studies have demonstrated that activation of PPAR*γ* does not promote lipid accumulation in either mouse or human macrophages [46–48]. Liver X receptor *α* (LXR*α*) is an oxysterol receptor that promotes cholesterol excretion and efflux by modulating expression of ATP-binding cassette transporter 1 (ABCA1) [47, 48]. LXR*α* was recently identified as a direct target of PPAR*γ* in mouse and human macrophages [49, 50]. Although the PPAR*γ*-induced increase in CD36 expression might accelerate lipid uptake in macrophages, subsequent activation of LXR*α* and upregulation of ABCA1 appear to induce lipid efflux. Diep et al. have demonstrated that rosiglitazone and pioglitazone attenuate the development of hypertension and structural abnormalities, and improve endothelial dysfunction in AngII-infused rats [51]. These TZDs also prevented upregulation of AT1R, cell cycle proteins, and inflammatory mediators. Rosiglitazone, but not the PPAR*α* ligand fenofibrate, prevented hypertension and endothelial dysfunction in DOCA-salt hypertensive rats [52]. It has been reported that serum levels of the soluble CD40 ligand are elevated in acute coronary syndrome and associated with increased cardiovascular risk. Treatment with rosiglitazone decreased the serum levels of soluble CD40 and MMP-9 in type 2 diabetic patients with coronary artery disease [53]. Taking all the evidence together, PPAR*γ* ligands may prevent the progression of atherosclerotic lesions, particularly in patients with DM (Figure 1).

## 3. Role of PPAR*γ* in Ischemic Heart Disease

Ischemic heart disease (IHD) is a disease characterized by ischemia to the heart muscle, usually due to coronary artery disease (atherosclerosis of the coronary arteries). Prolonged ischemia leads to cardiomyocyte death which is followed by a series of structural and functional alterations in the viable myocardium, known as cardiac remodeling. Adaptive changes in the extracellular matrix and in cardiomyocyte biology occur, which are initially able to maintain contractile function. However, progressive cardiac remodeling leads to chamber dilatation, contractile dysfunction and ultimately heart failure [54, 55]. Infiltration of neutrophils and macrophages is known to enhance the inflammatory response to myocardial ischemia and in combination with rapid accumulation of ROS within the ischemic zone can lead to tissue necrosis upon reperfusion [56]. This is further amplified by activation of redox-sensitive transcription factors, such as NF-*κ*B and AP-1, which control the expression of proinflammatory mediators, such as IL-12 and TNF*α*. Indeed, in an experimental rat model, inhibition of NF-*κ*B has been demonstrated to reduce reperfusion injury after a brief period of ischemia [57]. Furthermore, upregulation of AP-1 has been observed in cardiomyocytes in the presence of increased levels of ROS [58], such as those observed during ischemia and reperfusion, suggesting that this transcription factor may be involved in the pathogenesis of ischemia and subsequent reperfusion.

As the effects of PPAR*γ* on the heart are not fully understood, we and others have examined whether PPAR*γ* is involved in various heart diseases. Although the expression of PPAR*γ* in cardiac myocytes is low compared with adipocytes, PPAR*γ* ligands seem to act on cardiac myocytes [11, 59]. We demonstrated that PPAR*γ* ligands inhibited the cardiac expression of TNF-*α* at the transcriptional level, in part by antagonizing NF-*κ*B and AP-1 activity [11, 60]. Because TNF-*α* expression is elevated in the failing heart and has a negative inotropic effect on cardiac myocytes, treatment with PPAR*γ* ligands may prevent the development of CHF. Diabetic cardiomyopathy, which is characterized by systolic and diastolic dysfunction, is a major complication DM, and therefore TZDs seem to be beneficial for the impaired cardiac function in patients with DM. Following our study, the role of PPAR*γ* in myocardial ischemia-reperfusion (IR) injury has been elucidated [61–64]. In animal models, PPAR*γ* ligands reduced the size of the myocardial infarct and improved contractile dysfunction after IR through inhibition of the inflammatory response. IR injury activates JNK, and subsequently JNK induces increases in both AP-1 DNA-binding activity and apoptotic cells. It has been shown in rats that rosiglitazone inhibits the activation of JNK and AP-1 after myocardial IR [62]. Furthermore, pioglitazone has been reported to attenuate left ventricular remodeling and heart failure after myocardial infarction (MI) in mice [65]. Both of these effects of TZDs ligands were associated with decreases in inflammatory cytokines and chemokines [65, 66]. 

## 4. Role of PPAR*γ* in Cardiac Hypertrophy

Cardiac hypertrophy is characterized by maladaptive changes in myocardial structure and function, which are collectively known as cardiac remodeling. Cardiac hypertrophy is an independent risk factor for heart failure, arrhythmia, and sudden death and is one of the most potent predictors of adverse cardiovascular outcomes in hypertensive patients [67, 68]. Initially, the heart compensates for the increased wall stress by undergoing significant alterations in cardiomyocyte biology and in the extracellular matrix. However, progressive LV hypertrophy combined with loss of collagen crosslinking and myocyte slippage causes increased wall stress leading to cardiac chamber dilatation, contractile dysfunction, and ultimately decompensated congestive heart failure (CHF) [69]. Changes in structure and function of the heart are mediated by a variety of mechanical, neuronal and hormonal factors. Several antihypertensive drugs such as angiotensin-converting enzyme (ACE) inhibitors, angiotensin receptor blocker (ARB), and *β*-blockers are known to attenuate cardiac remodeling and have morbidity/mortality benefits [70, 71], but its effect is not enough.

The PPAR*γ* ligands, troglitazone, pioglitazone, and rosiglitazone, inhibited AngII-induced hypertrophy of neonatal rat cardiac myocytes [72–74]. Because generalized PPAR*γ* gene deletion causes embryonic lethality, we examined the role of PPAR*γ* in the development of cardiac hypertrophy in vivo using heterozygous PPAR*γ*-deficient (PPAR*γ* ^+/-^) mice [72]. Pressure overload-induced cardiac hypertrophy was more prominent in heterozygous PPAR*γ* ^+/-^ mice than in wild-type (WT) mice. Treatment with pioglitazone strongly inhibited the pressure overload-induced cardiac hypertrophy in WT mice and moderately in PPAR*γ* ^+/-^ mice [72]. Thereafter, 2 other groups examined the role of PPAR*γ* in the heart by using cardiomyocyte-specific PPAR*γ* knockout mice [75, 76]. Duan et al. reported that these mice develop cardiac hypertrophy through elevated NF-*κ*B activity [75], and unexpectedly, rosiglitazone-induced cardiac hypertrophy in both the WT mice and cardiomyocyte-specific PPAR*γ* knockout mice through activation of p38 MAP kinase independent of PPAR*γ*. Ding et al. reported that cardiomyocyte-specific PPAR*γ* knockout mice displayed cardiac hypertrophy from approximately 3 months of age and then progress to dilated cardiomyopathy (DCM), and most mice died from heart failure within 1 year after birth [76]. Mitochondrial oxidative damage and reduced expression of manganese superoxide dismutase were recognized in the cardiomyocyte-specific PPAR*γ* knockout mice [76]. These mice models demonstrate that PPAR*γ* is essential for protecting cardiomyocytes from stress and oxidative damage, although the expression level of PPAR*γ* in cardiomyocytes is low. On the other hand, Son et al. demonstrated that cardiomyocyte-specific PPAR*γ* transgenic mice develop DCM associated with increased uptake of both fatty acid and glucose [77]. Rosiglitazone increased this glucolipotoxicity in cardiomyocyte-specific PPAR*γ* transgenic mice. If PPAR*γ* in the heart is expressed at a high level, rosiglitazone may cause cardiotoxic effects; however, as noted earlier the expression level of PPAR*γ* in the heart is quite low. Because cardiac hypertrophy can be seen even in normotensive diabetic patients, and diabetic cardiomyopathy is a major complication of DM, antidiabetic agents such as the TZDs would be expected to have beneficial effects on cardiac hypertrophy and dysfunction in patients with DM.

## 5. Role of PPAR*γ* in Myocarditis

Myocarditis is a potentially life-threatening disease that primarily affects children and young adults with sometimes devastating consequences, including sudden death. The primary long-term consequences are DCM and CHF. Myocarditis presents with a spectrum of symptoms ranging from mild dyspnea or chest pain that spontaneously resolves without treatment to cardiogenic shock and sudden death. The major long-term consequence is DCM with CHF. Common viral infections are the most frequent cause of myocarditis, but other pathogens, hypersensitivity reactions, and systemic and autoimmune diseases have also been implicated.

**Figure 2:**
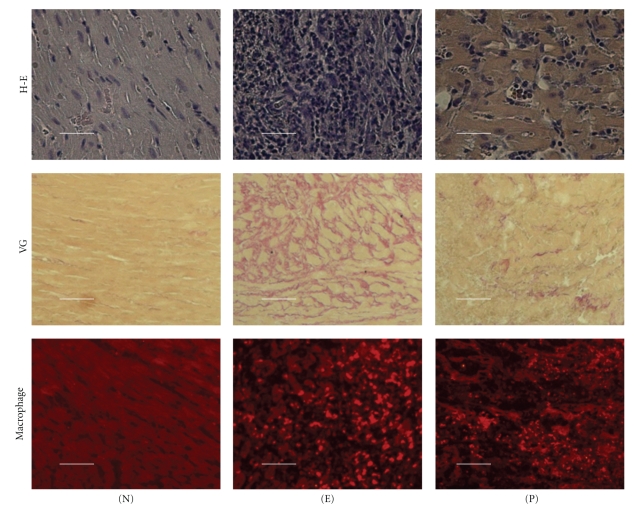
Histopathology of the heart section of experimental myocarditis [78]. (N); control heart, (E); heart from experimental myocarditis treated with saline, (P); heart from experimental myocarditis treated with pioglitazone. The upper panel shows hematoxylin-eosin staining (scale bars indicate 200 *μ*m), the middle panel shows van Gieson staining (scale bars indicate 400 *μ*m), and the lower panel shows immunostaining of infiltration of inflammatory macrophages (scale bars indicate 200 *μ*m) in the heart of experimental myocarditis rats. The degree of inflammation, fibrosis and infiltration of inflammatory macrophages was significantly improved by the treatment with pioglitazone.

**Figure 3:**
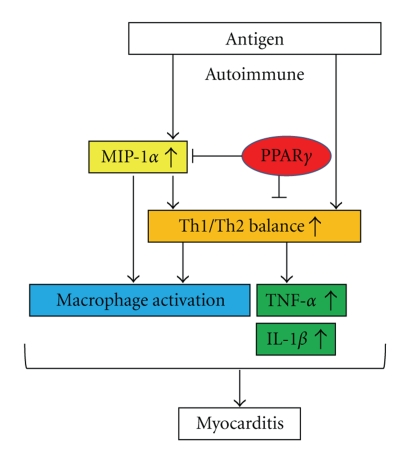
Inhibitory mechanisms of PPAR*γ* on autoimmune induced myocarditis. PPAR*γ* inhibits the progression of autoimmune induced myocarditis via inhibition of the activation of Th1/Th2 balance, MIP-1*α*, proinflammatory cytokines TNF-*α*/IL-1*β*, and macrophage.

Rat experimental autoimmune myocarditis (EAM) model is a T cell-mediated disease characterized by infiltration of T cells and macrophages, leading to massive myocarditis necrosis, which develops into heart failure in the chronic phase [79]. Two weeks after immunization with porcine cardiac myosin, small numbers of CD4^+^ T cells and macrophages start to infiltrate into the myocardium and various cytokines are expressed. Macrophage inflammatory protein-1*α* (MIP-1*α*) is a C-C chemokine that induces leukocyte accumulation in tissue sites of inflammation. We previously demonstrated that MIP-1*α* mRNA and protein are highly expressed in the hearts of rats with EAM from day 11 after first immunization (Figure 2) [79]. MIP-1*α* drives naive helper T (Th) cells to differentiate into type 1 helper T (Th1) cells in the early stage, and thereafter several cytokines are secreted by Th1 cells or macrophages. Th1 cells produce interferon-*γ* (IFN-*γ*), which is mainly involved in cell-mediated immune responses, whereas type 2 helper T (Th2) cells produce IL-4, IL-5, IL-6, IL-10, and IL-13, which participate in humoral responses. Immune dysfunction associated with autoimmune disease is known to involve an imbalance between Th1 and Th2 cells. We and others have reported that pioglitazone treatment markedly reduces the severity of myocarditis in a rat model of EAM [78, 80]. Pioglitazone suppressed expression of inflammatory cytokines and activation of myocardiogenic T cells in the myocardium of EAM rats [80]. The mRNA levels of MIP-1*α* were upregulated in the hearts of EAM rats, but not in the hearts of those in the pioglitazone group. The expression of inflammatory cytokines such as IL-1*β* and TNF-*α* was upregulated in EAM rats and it was reduced by the treatment with pioglitazone. Furthermore, treatment with pioglitazone decreased the Th1 cytokine (IFN-*γ*) genes and increased the expression levels of Th2 cytokine (IL-4) gene [78]. These results suggest that PPAR*γ* ligands change the orientation of immune responses by favoring Th2 response. The treatment with PPAR*γ* ligands may have beneficial effects on myocarditis by inhibiting MIP-1*α* expression and modulating the Th1/Th2 balance (Figure 3). Further studies are necessary to elucidate whether PPAR*γ* ligands have beneficial effects on human myocarditis.

## 6. Clinical Efficacy and Safety of TZD Treatment

TZDs (or glitazones) are widely used in the treatment of type II diabetes, respectively. Although these are their primary indications due to positive effects on glucose homeostasis, atherogenic proteins, endothelial function, and inflammation, these compounds may also be of benefit in other related pathologies, such as CVD [81]. There are currently two TZDs in clinical use, rosiglitazone and pioglitazone. The first agent in this class (troglitazone) was withdrawn after reports of hepatic toxicity and failure. Clinical studies have shown that TZDs improve insulin resistance and lower blood glucose levels in subjects with Type 2 diabetes. Growing evidence support the concept that rosiglitazone and pioglitazone have beneficial effects on several cardiovascular risk factors and surrogate markers, beyond their effects on glycemic control and the clinical significance of the beneficial effects of TZDs on CVD has been clarified [82].

Two studies demonstrated that treatment with pioglitazone inhibit the progression of carotid intima/medial thickness (IMT) and coronary atheroma volume, which is important surrogates of atherosclerosis [83, 84]. PROspective pioglitAzone Clinical Trial In macroVascular Events (PROactive) trial, which was a secondary prevention trial in patients with type 2 diabetes and preexisting CVDs (previous MI, stroke or peripheral vascular disease), was the first cardiovascular outcome study with a TZD to be published [85]. It was a randomized double-blind trial comparing the efficacy of pioglitazone in reducing the incidence of new macrovascular events or death with placebo in 5238 patients with type 2 diabetes and macrovascular disease. The primary endpoint was the composite of all-cause mortality, nonfatal MI, stroke, ACS, endovascular or surgical intervention in the coronary or leg arteries, and amputation above the ankle. Pioglitazone treatment resulted in a nonsignificant 10% reduction (HR 0.90; *P* = .095) in the primary composite endpoint and a significant 16% reduction (HR 0.94; *P* = .027) in the main secondary endpoint of all-cause mortality, nonfatal MI, and stroke combined, in comparison with placebo, after a mean followup of about 34.5 months. Although there was a 1.6% absolute increase in heart failure hospitalizations in the pioglitazone group compared with the placebo group, the number of heart-failure-related deaths was almost identical. In subgroup analysis (PROactive 05), pioglitazone treatment resulted in a 28% reduction (HR 0.82; *P* = .04) of fatal and nonfatal MI and a significant 37% reduction (HR 0.67; *P* = .035) of acute coronary syndrome in patients who had experienced a previous MI six months or more before randomization [86]. Moreover, in patients who had experienced a previous stroke six months or more before randomization, there was a significant 47% reduction (HR 0.53; *P* = .0085) of fatal or nonfatal stroke and a marginally significant 28% reduction (HR 72; *P* = .0467) of the composite end point of cardiovascular death, nonfatal MI, or nonfatal stroke (PROactive 04) [87]. In meta-analysis, pioglitazone has been reported not to associate with increased risk of either MI or CV mortality [88, 89].

Meanwhile, it has been reported that rosiglitazone treatment is associated with overall increased incidence of MI (OR 1.43; *P* = .03) and a potential increase of borderline significance in the risk of death from total CV causes (OR 1.64; *P* = .06) by meta-analysis [90]. Several analyses have been performed that challenged these findings, but the CVD risk of rosiglitazone is uncertain [91–93]. Another meta-analyses of RCTs of rosiglitazone in patients with type 2 diabetes showed that rosiglitazone significantly increased the risk of MI (RR 1.42; *P* = .02) and heart failure (RR 2.09; *P* < .001), but not the risk of cardiovascular mortality (RR 0.90; *P* = .53) [94]. Conversely, a pooled analysis of 3 large rosiglitazone trials designed to specifically test CV outcomes (a diabetes outcome progression trial, rosiglitazone evaluated for cardiac outcomes and regulation of glycemia in diabetes, and diabetes reduction assessment with ramipril and rosiglitazone medication) did not reach statistical significance for either MI (OR 1.29; *P* = .12) or death due to CV causes (OR 0.90; *P* = .67) [95]. Furthermore, a meta-analysis of 86 trials did not find a statistically significant increase in the overall rate of MI in patients on rosiglitazone [96]. A further study is needed to establish the utility of rosiglitazone in CVD.

There are some differences in the actions of pioglitazone and rosiglitazone. Differences in side chains are responsible for differences between the compounds in pharmacodynamic and pharmacokinetic properties, as well as the side-effect profiles. Pioglitazone has more beneficial effects on the lipid profile than rosiglitazone [97]. As mentioned earlier, rosiglitazone, but not pioglitazone, induced cardiac hypertrophy by a non-PPAR*γ*-mediated pathway [75]. Pioglitazone represses NF-*κ*B activation and VCAM-1 expression in a PPAR*α*-dependent manner [98]. Pioglitazone was recently reported to increase the number and function of endothelial progenitor cells (EPCs) in patients with stable coronary artery disease and normal glucose tolerance [99]. Pioglitazone may induce angiogenesis by modulating EPCs mobilization and function. In the future, more mechanistic studies are required to investigate the differences in action between pioglitazone and rosiglitazone.

TZDs do not directly affect left ventricular systolic or diastolic function and may even be beneficial [100]. However, data from the recently published meta-analyses indicate that treatment with TZDs significantly increase the risk of CHF (RR 1.72; *P* = .002) [101]. Both pioglitazone and rosiglitazone unambiguously increase risk of CHF, potentially reflecting an inherent proclivity to induce edema [102]. Pioglitazone and rosiglitazone appeared to raise the risk of CHF by similar amounts, but neither raised the incidence of heart-failure-associated death [101]. Clinical studies report TZD-induced peripheral fluid retention, and an increase in plasma volume in 2–5% of patients on monotherapy [103]. The exact mechanisms for TZD-induced fluid retention are not well understood, and it remains unclear whether TZDs directly cause the development of de novo CHF. Fluid retention was more likely to occur with concomitant insulin use, and in patients with underlying cardiac dysfunction or renal insufficiency. The level of vascular endothelial growth factor is increased in the patients who develop fluid retention with TZD therapy and this may lead to peripheral edema through increased vascular permeability [104]. The insulin-sensitizing action of TZDs also induces water and salt retention. PPAR*γ* is highly expressed in the kidney and collecting-duct-specific PPAR*γ* knockout mice demonstrated no effects of TZD on fluid retention or the expression level of sodium channel ENaC-*γ* [105, 106]. These findings suggest that activation of the sodium channel in the collecting duct cells expressing PPAR*γ* may be a mechanism of fluid retention. In patients without evidence of heart failure, careful examination did not reveal any worsening of left ventricular function by TZDs [107]. Of note, diabetes itself affects both diastolic and systolic functions and is an independent risk factor for the development of CHF [108]. The American Heart Association (AHA) and American Diabetes Association (ADA) have released a consensus statement that advises caution regarding the use of TZDs in patients with known or suspected heart failure [108]. Because there is a possibility that TZDs may unmask asymptomatic cardiac dysfunction by increasing plasma volume, they should be avoided in patients with CHF of New York Heart Association (NYHA) class III or IV.

Angiotensin II receptor blockers (ARBs) are widely used for the treatment of hypertension, ischemic heart disease, and heart failure. ARBs have been reported to have protective effects on the cardiovascular system beyond blood pressure lowering effect in many clinical trials [109]. Telmisartan, one of the ARBs, has recently been identified as a partial activator of PPAR*γ*. There is evidence that, in contrast to some other ARBs, telmisartan may exert beneficial effects on insulin sensitivity through activation of PPAR-*γ* [110]. There is a structural resemblance between telmisartan and PPAR-*γ* ligand pioglitazone, which suggests that telmisartan may activate the receptor. In vitro studies showed that telmisartan acted as a partial agonist of PPAR-*γ* and modulated the expression of PPAR-*γ* target genes involved in carbohydrate and lipid metabolism [111]. Telmisartan induces adiponectin expression via PPAR*γ* activation [112], and adiponectin has been reported to induce angiogenesis [113]. Telmisartan-induced proliferation of human EPCs via PPAR*γ*-dependent PI3K/Akt signaling pathway [114]. Since telmisartan has both effects of angiotensin II blockade and PPAR*γ* activation as well as getting out from under the condition of vascular dysfunction, it might be expected to recover vascular function and promote neoangiogenesis in the ischemic tissue via proliferation of EPCs beyond the class effects of ARBs in the clinical setting. In the Ongoing Telmisartan Alone and in Combination with Ramipril Global Endpoint Trial (ONTARGET), telmisartan was reported to be as effective as ramipril for the primary cardiovascular outcome during a 56-month followup but was better tolerated [115]. The utility of telmisartan by having the additional value of PPAR*γ* activity is expected.

## 7. Conclusions

Since the discovery of nuclear PPAR*γ* in the early 1990s, it becomes evident that PPAR*γ* play an important role in the cardiovascular system and implicate in several CVDs. The data from in vitro studies suggest that TZDs exert direct actions on vascular cells and cardiomyocytes, independent of their glucose-mediated mechanisms. The subsequent development of PPAR*γ* agonists and gene-modified animals has highlighted the involvement of these receptors in numerous biological pathways. However, the complexity of PPAR*γ* activation, in combination with their diverse tissue distribution and lack of specificity of currently available PPAR*γ* agonists makes therapeutic modification of PPAR*γ* pathways a challenging goal. A detailed understanding of the role of PPAR*γ* in the cardiovascular system is required in order to delineate the precise mechanisms by which PPAR*γ* may modify cellular CVD processes and enable identification of effective therapeutic targets. Further studies using tissue-specific gene targeting mice are necessary to address the pleiotropic effects of PPAR*γ* on the cardiovascular system. Future research aimed at the development of more effective agonists. Dual PPAR*α*/*γ*, PPAR*δ*/*γ* agonist, pan PPAR*α*/*δ*/*γ* agonists, and combination of PPAR*γ* with other cardiovascular drugs will address some of the issues currently surrounding the potential use of PPAR*γ* activators in the treatment of CVD.
